# Identification of RPGRIP1L as an instability-maintaining gene to drive tumor growth and PD-L1 expression via Hedgehog signaling in breast cancer

**DOI:** 10.1186/s12885-025-15500-2

**Published:** 2025-12-30

**Authors:** Meng Lv, Yan’e Liu, Weihong Cao, Yongmei Wang, Qi Wang, Xueqiang Gao, Baowei Peng, Haibo Wang, Yan Mao

**Affiliations:** 1 Breast Disease Center, The Affiliated Hospital of Qingdao University, No 59 Haier Road, Qingdao, Shandong 266003 China; 2 Department of Medical Oncology and Radiation Sickness, Peking University Third Hospital, Peking University, Beijing, P. R. China; 3 Department of Radiation Oncology, the Affiliated Hospital of Qingdao University, Qingdao, Shandong 266003 P. R. China; 4 College of Pharmacy, Dali University, Dali, 671003 Yunnan China

**Keywords:** Breast cancer, Genome instability, Hedgehog signaling, Immune checkpoints, RPGRIP1L

## Abstract

Genomic instability is a hallmark of nearly all human cancers, yet the genetic regulators that govern this instability, their functional roles, and their therapeutic potential remain incompletely understood in breast cancer. In this study, we conducted a comprehensive analysis of the expression profiles of genomic instability-maintaining genes (GIMGs) in breast cancer and revealed that elevated expression of GIMGs was associated with adverse clinical outcomes and an altered tumor immune microenvironment. Through further analysis, we identified a core set of GIMGs and pinpointed RPGRIP1L as a critical driver of genomic instability in breast cancer. Notably, RPGRIP1L expression was significantly upregulated in breast cancer tissues and strongly correlated with poor patient prognosis. Functional studies demonstrated that RPGRIP1L knockdown reduced genomic instability in tumor cells, as evidenced by decreased Phosphorylated Histone H2AX (γH2AX) expression, and suppressed tumor growth in mouse models. Additionally, high RPGRIP1L expression was linked to elevated expression of immune checkpoint Programmed Death Ligand 1(PD-L1) in human breast cancer samples. Mechanistically, we found that RPGRIP1L promoted the activation of the Hedgehog signaling pathway, which in turn drived tumor proliferation and upregulated PD-L1 expression. Collectively, these findings highlight RPGRIP1L as a key genomic instability-maintaining gene in human breast cancer, offering critical insights into the molecular mechanisms underlying disease progression. Furthermore, targeting RPGRIP1L may represent a promising therapeutic strategy for breast cancer.

**Supplementary Information:**

The online version contains supplementary material available at 10.1186/s12885-025-15500-2.

## Introduction

Genome integrity is important for proper function of organism, but constantly challenged by external or internal stressors and lead to gene mutations and damage in the chromosomes [1]. Defects in safe guard mechanisms maintaining genome integrity when responding to DNA damage [2] or mitotic chromosomal imbalances [3] lead to the accumulation of mutations, which is known as genome instability. Genome instability will affect gene expression and the function of proteins. It will induce phenotypic changes in cells, which trigger the development of diseases, such as cancer, inflammatory-associated diseases and ageing [4–7]. A network of various signaling pathways including cell cycle checkpoints, DNA repair, recombination and apoptosis effectively have been reported to maintain the integrity of the human genome [8]. It is estimated that 60%–80% of human tumors exhibit chromosomal abnormalities suggestive of chromosomal instability (CIN) [9, 10]. CIN positively correlates with tumor stage and is enriched in relapsed as well as metastatic tumor specimens [11–14]. Although genomic instability is a characteristic of human cancers, knowledge of the genetic factors maintain genomic instability is largely unknown. Clarifying the mechanisms controlling the instability of genomes can reveal central insights about how defects in these processes cause genomic instability and important for understating the mechanism for tumor initiation and progression.

Breast cancer is the most frequently occurring type of cancer and also the primary cause of cancer related death in female around the world [15]. Breast cancer is a diverse disease with high heterogeneity, and manifests in distinct clinical and pathologic features. Generally, five intrinsic molecular subtypes of breast cancer, including Luminal A, Luminal B, HER2-overexpression, Basal-like and Claudin-low have been identified according to the expression of ER, PR, Her-2 and Ki-67 [16]. Genomic instability is a hallmark of tumorigenesis in breast cancer. However, owing to the high complexity of breast tumors, the genomic instability may occur in distinct patterns and can predict prognosis for the patient. Understanding the mechanisms controlling the genomes stability in breast cancer may benefit for accurate diagnosis and precision treatment in breast cancer patients. However, the dysregulated genes controlling the genomes stability, and the genetic determinants of genomic instability in different subtypes of breast cancer are unknown.

By screening almost 1,000 mouse mutants, Adams et al. have identified 145 genes regulating genome stability that modified micronucleation in both mouse and human cells, including 74 genes promoting genome instability [17]. To explore the genome instability regulating genes in breast cancer, here, we interrogated the 74 genes and performed systematical analysis to catalogue the genetic elements in regulating genome stability in breast cancer. Our study aims to provide a novel resource that highlights specific gene sets linked to genomic instability in breast cancer, while validating the functional role of a key gene in driving tumor progression in this context.

## Materials and methods

### Clinical specimens

Breast cancer specimens were obtained from Breast Disease Center, The Affiliated Hospital of Qingdao University. All clinical samples were collected with informed consent in accordance with International Ethical Guidelines for Biomedical Research Involving Human Subjects. The study was approved by the Research Ethics Committee of Qingdao University (QYFYKYLL 915811920).

### Animal experiments

Five-week-old C57BL/6 female mice were purchased from Cyagen Co., Ltd and kept on a 12-h day/night cycle and fed a normal chow diet. To generate a orthotopic xenograft model, 1 × 10^6^ EO771 tumor cells were resuspended in culture medium containing 50% Matrigel. The suspension was then orthotopically implanted by injection into the 4th mammary fat pad. At week 4, the mice were humanely euthanized by cervical dislocation after deep anesthesia with isoflurane, and tumors were collected, weighted, measured, fixed and prepared for the histological examination. All animal experiments were conducted in accordance with the National Institutes of Health Guide for the Care and Use of Laboratory Animals. All animal studies were approved by the Institutional Animal Care and Committee of Dali University (2022-PZ-24).

### Cell culture and reagents

Murine breast cancer cell line EO771 tumor cells were cultured in medium according to the standard protocols, supplemented with 10% FBS, 100 units/mL penicillin and 100 µg/mL streptomycin. Cells were treated with GANT 61 (20 µM, # HY-13901, MCE), or cultured under hypoxic conditions for 4 h (1% O2, 5%CO2 and 94%N2).

### Cell counting kit-8 (CCK8)

CCK8 (#SB-CCK8, ShareBio) was performed following the instruction.

### Immunohistochemistry

Immunohistochemistry (IHC) were performed routinely. Briefly, the paraffin-embedded slices (5 μm thickness) were deparaffinized and rehydrated according to standard protocols. After blocking in 10% BSA, the sections were incubated with primary antibodies Ki67 (1:100, Servicebio, #GB111499) or RPGRIP1L (1:50, Wuhan Fine, #FNab07404) overnight at 4 °C. On the second day, the sections were incubated with HRP conjugated secondary antibodies for 45 min at room temperature, and then stained with DAB substrate and counterstained by hematoxylin. The pathology scores of RPGRIP1L were assessed independently by two senior pathologists in a blinded manner, based on the percentage of positively stained cells and the staining intensity as previously reported [18]. Scores based on the percentage of positive cells: 0 (0–5% positive cells), 1 (5%-35%), 2 (35%-70%) and 3 (> 70%). Scores based on the staining intensity: 0 (no), 1 (week), 2 (modest) and 3 (strong). The final score was calculated by multiplying the percentage and intensity scores: “-” (0–1), “+” (2–3), “++” (4–6), and “+++” (> 6). Notably, Low expression:<4, high expression:≥4.

### Lentivirus production and cell transfection

We constructed shRNA plasmids targeting mouse RPGRIP1L, and lentivirus particles were generated in 293T cells. Target cells were incubated with the lentivirus encoding shRNA for 8 h, followed by selection with 2.5 mg/mL puromycin beginning 48 h post-infection and continuing for 5 days. The knockdown efficiency of RPGRIP1L in puromycin-resistant cells was assessed by real-time quantitative PCR and Western blotting.

### Western blotting

Cells were lysed using protein lysis buffer (#P70100, NCM Biotech) with protease inhibitor cocktails (#P002, NCM Biotech) and separated by SDS-PAGE gel electrophoresis and transferred to Nitrocellulose membranes (#10600001, Cytiva). The membranes were blocked with 5% skim milk for 1 h and then incubated overnight at 4 °C with one of the following primary antibodies: RPGRIP1L (1:1000), GLI1 (1:1000, 66905-1-Ig, Wuhan, Proteintech), anti-beta-actin (1:3,000, #AB0035, Abways, Shanghai, China). After incubated with HRP-conjugated secondary antibodies for 1 h at room temperature and visualized using an ECL chemiluminescence assay.

### Quantitative real-time PCR

Total RNA was isolated using trizol and reverse transcription was performed. Subsequently, Real-time PCR analyses were conducted on a 7500 real-time qPCR system from Applied Biosystems. Relative mRNA expression was calculated by normalizing with β-actin gene expression, applying the 2^(−ΔΔCt)^ method. The specific sequences of primers were: RPGRIP1L, forward GCCGGTGAAAGATACAGGTCT, reverse ACGCAAAAATCTGTCTTCCAGT; CD247: forward GGGAGGCAAACAGAGGAGG, reverse CTGGGAGGCTAAGAGGCTTC.

### Data sources

RNA-seq data and clinicopathological information for BRCA patients were collected from two different sources: The Cancer Genome Atlas (TCGA)-BRCA dataset (https://portal.gdc.cancer.gov/) and the GSE7390 dataset (https://www.ncbi.nlm.nih.gov/geo). The samples without clinical data were excluded. Additionally, the scRNA-seq data was obtained using TISCH database (http://tisch1.comp-genomics.org) based on GSE136206 dataset. The RNA-seq data from breast cancer patients GSE162228, GSE20711, GSE21653, GSE25055, GSE48390 was obtained from BEST (https://rookieutopia.com/).

### Construction of the Genomic Instability-Maintaining Genes (GIMGs) signature in breast cancer

In the TCGA-BRCA dataset, we initially conducted a univariate Cox regression analysis to identify key genes with prognostic significance (*p* < 0.05) for constructing a risk model based on GIMGs. Subsequently, a Least Absolute Shrinkage and Selection Operator (LASSO) regression analysis was applied to refine the risk model. The risk score was derived from the expression of DSCC1, RPGRIP1L, and IRF1, weighted by their regression coefficients from the LASSO-Cox model. The formula for the risk score was then derived as follows:

$$\mathrm{riskscore}=\sum\left[\mathrm{Exp}\left(\mathrm{gene}\right)\times\mathrm{coef}\left(\mathrm{gene}\right)\right]$$

Here, Exp(gene) represents the gene expression levels, and coef(gene) denotes the regression coefficient. The ‘survminer’ and ‘survival’ packages were utilized to visualize the receiver operating characteristic (ROC) curves and calculate the areas under the time-dependent ROC curves (AUCs). Additionally, the validation of the risk model was performed using the GSE7390 dataset.

### Consensus unsupervised clustering

The unsupervised cluster analysis was realized by ‘ConsensusClusterPlus’ R package and the ‘K-Means’ algorithm was applied. Then a measure of distance was represented by ‘euclidean’. The proportion of ambiguous clustering was used to determine the optimal k value. After that, the differentially expressed genes (DEGs) between the two clusters were identified by the ‘limma’ R package with a criterion of |log2(foldchange)|>1 and adjust *p* < 0.05.

### Gene Ontology (GO), Kyoto Encyclopedia of Genes and Genomes (KEGG) Enrichment Analysis and Gene Set Enrichment Analysis (GSEA)

The differentially expressed genes (DEGs) were filtered at a criterion of |log2(foldchange)|>1 and adjust *p* < 0.05. GO and KEGG enrichment analysis were conducted by the ‘clusterProfiler’, ‘enrichplot’ and ‘org. Hs.e.g.db’ packages. GSEA based on GO and KEGG gene sets was also implemented.

### To construct the nomogram and calibration curves

Principal component analysis (PCA) was utilized to evaluate the grouping ability of the risk score. A nomogram was developed incorporating the risk score and various clinicopathological characteristics to predict the overall survival rates of patients with breast cancer at 1, 3, and 5 years. The accuracy of the predictions was evaluated using calibration curves.

### Immune related analysis

The CIBERSORT algorithm was used to reveal the fraction of infiltrating immune cells in breast cancer samples from TCGA, and the “corrplot” package was utilized to explore the correlation among various immune cells. Furthermore, the ‘ESTIMATE’ package was used to calculate the tumor purity and immune scores initially. The expression levels of HLA family genes and immune checkpoint genes were compared between two groups.

### Tumor Mutational Burden (TMB)

The TMB for each sample was assessed, aggregated, and evaluated with the ‘maftools’ package. Waterfall plots were employed to visualize the mutational profiles, and survival analysis was conducted based on different TMB levels using the ‘survminer’ and ‘survival’ packages.

### Statistical analysis

All the bioinformatics analyses were analyzed in R (version 4.0.2; R). The numerical data presented in this study was analyzed by the GraphPad Prism 8.4.3. Student’s t-test was implemented for comparisons between two groups. The Log-rank test was utilized to compare the survival time in Kaplan-Meier survival curve. The results were considered statistically significant at *P* < 0.05.

## Results

### Identifying and establishing a Genomic Instability-Maintaining Genes (GIMGs) signature in The Cancer Genome Atlas (TCGA) Breast Invasive Carcinoma (BRCA) cohort

**Fig. 1:**
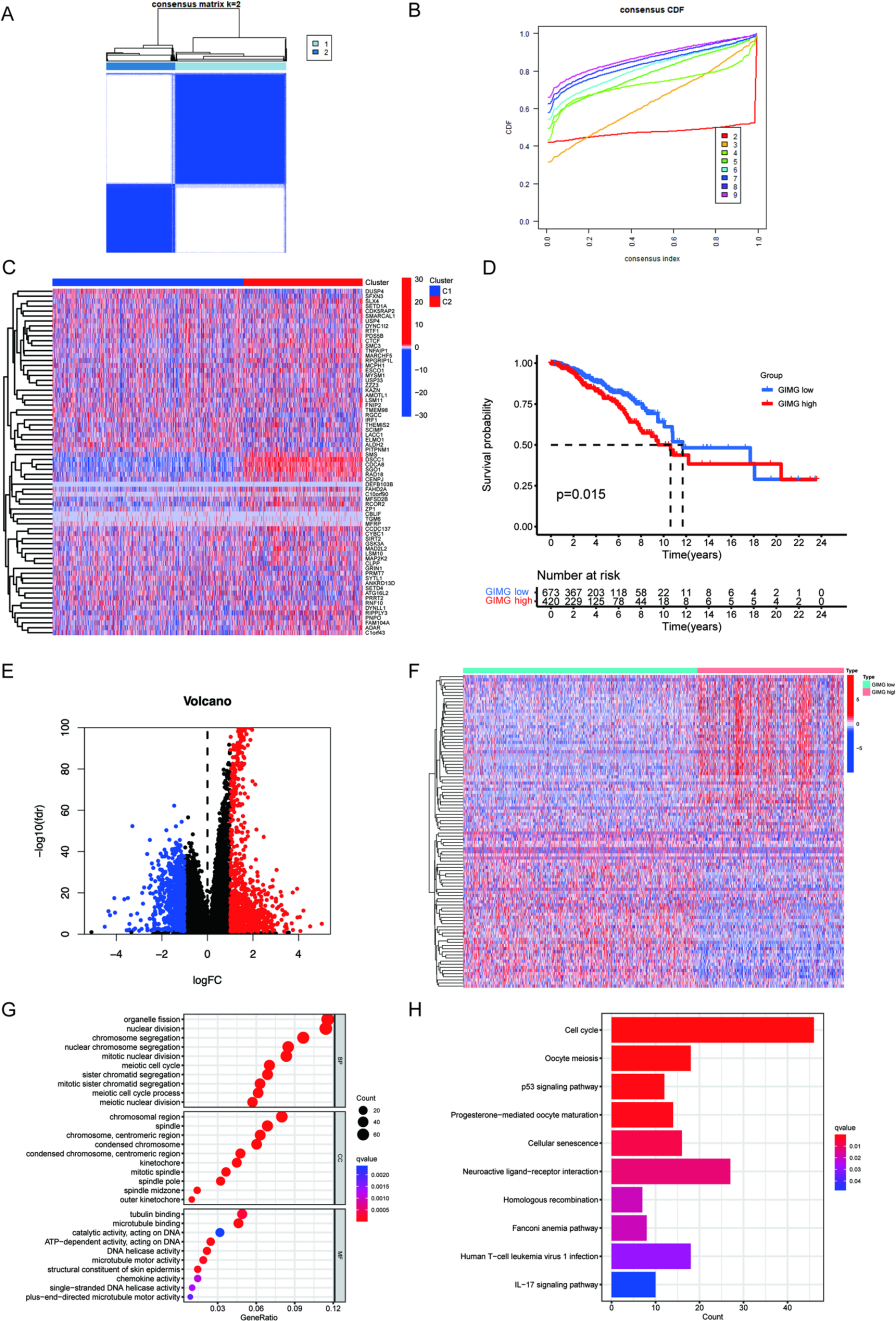
Enrichment analyses of differentially expressed genes between genomic instability-maintaining genes (GIMG)-high and low group. (**A)** Heatmap of consensus clustering solution (k = 2) in BRCA samples. (**B)** The delta area curve of consensus clustering. **C **and **D** The Cancer Genome Atlas (TCGA) RNA sequencing data of breast cancer were divided into two groups based on expression of genomic instability-maintaining genes (GIMGs) (C). Kaplan-Meier curve of OS between the two groups (**D**). **E** and **F**. Volcano chart (**E**) and heatmap (**F**) showing the differential expressed genes in the two groups (GIMG high and GIMG low). GO (**G**) and KEGG (**H**) enrichment analysis of increased expressed genes in GIMG high-expressing group compared with low-expressing group |FoldChange|> 1.5, adj *P* < 0.05

We first investigate the expression and prognostic value of genome instability maintaining genes (GIMGs) in breast cancer using The Cancer Genome Atlas (TCGA)-Breast invasive carcinoma (BRCA) cohort. The GIMGs were ascertained according to the published literature which systemically identified GIMGs in mice [17]. After matching their expression in Homo sapiens, we got 74 GIMGs which were used for analysis in the following work. The TCGA dataset of BRCA, which includes 1093 tumors and 113 normal tissues, along with all the clinical information was downloaded. By employing consensus unsupervised clustering, we discerned two distinct clusters, designated as C1 and C2, within the TCGA cohort (Fig. 1A-B). Subsequent differential analysis unveiled that, when compared to C1, the expression levels of most GIMGs were significantly higher in C2 (Fig. 1C). As a result, we classified C2 as the GIMG high cluster and C1 as the GIMG low cluster. BRCA patients with high-expression of GIMGs showed significantly shorter overall survival (OS) time than those with low-expression (Fig. 1D). Volcano and heap chart showed the expression of differential expressed genes in the two groups (Fig. 1E-F). Consistently, GO and KEGG enrichment analysis showed multiple pathways promoting tumor progression were enriched in the GIMGs-high group compared with the low group (Fig. 1G-H).

### GIMGs were associated with altered immune microenvironment and higher immune checkpoints expression in TCGA breast cancer cohort

**Fig. 2:**
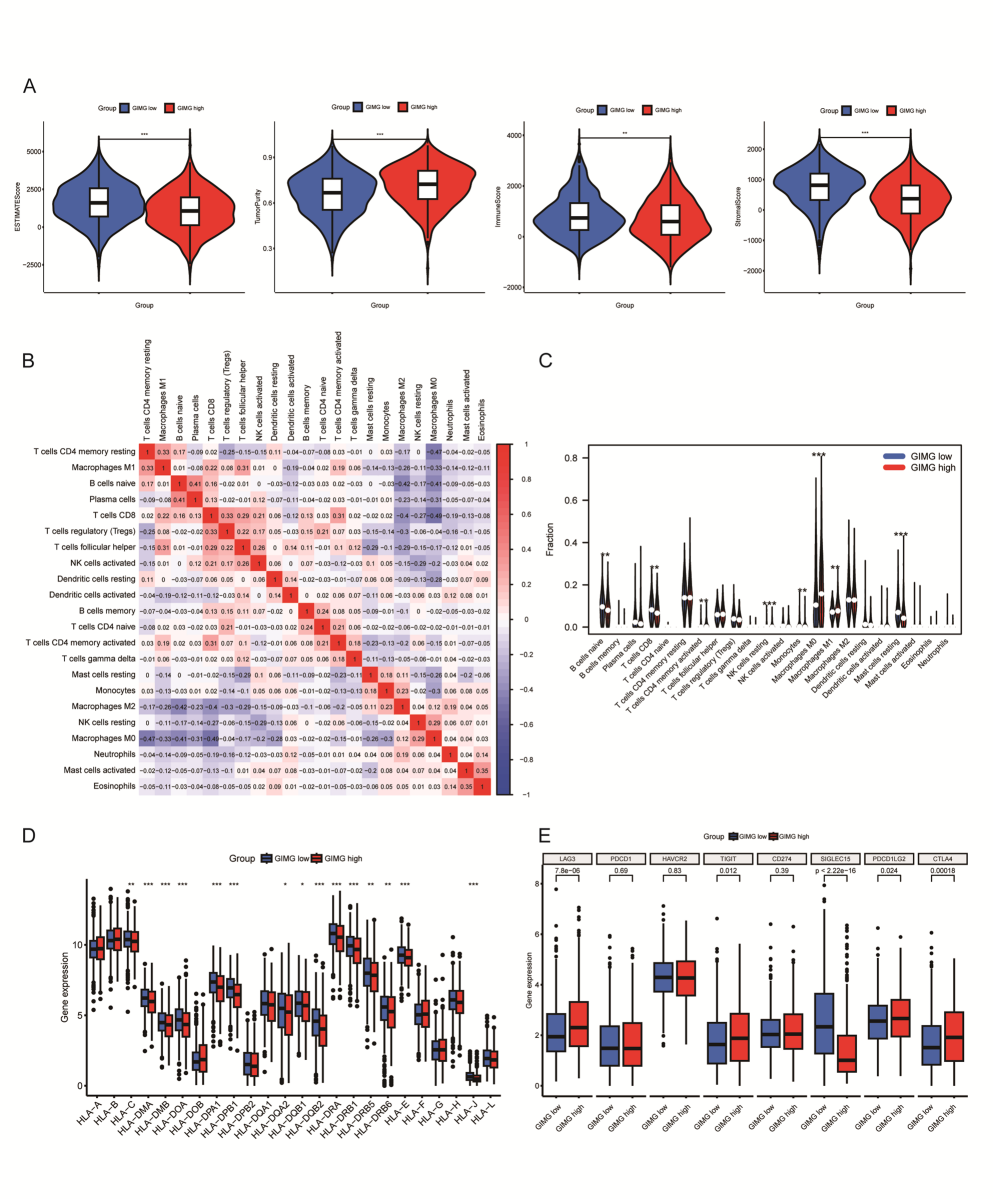
The difference of tumor immune microenvironment between GIMG high- and low- groups in breast cancer patients. (**A)** The graph showing the estimate score, tumor purity, immune score and stromal score between GIMG-low and GIMG-high group. **B**-**C**. The heatmap (**B**) and bar chart (**C**) revealed the infiltrating immune cells as calculated by CIBERSORT algorithm in the two groups. The expression of HLA family genes (**D**) and immune check points (**E**) in the two groups. ****p < 0.0001; ***p < 0.001; **p < 0.01; *p < 0.05

The tumor cell-intrinsic effect by genome instability potentiates affects tumor microenvironment [19].The immune scores and stroma scores are significantly lower in GIMGs-high group compared with low group in breast cancer samples from TCGA (Fig. 2A). Pearson’s correlation heatmap suggested that the correlation differed among immune cells (Fig. 2B). Notably, a significant positive correlation between activated CD4 + T memory cells, NK resting cells, M1 macrophage, while a negative correlation between B cells naïve, CD8 + T cells, monocytes, and resting mast cells was observed with GIMGs-high group (Fig. 2C). This was consistent with the previous scRNA-seq data in mice breast cancer. That result showed that chromosomal instability (CIN) high tumor cells were markedly enriched in immune-suppressive macrophages, granulocytic myeloid-derived suppressor cells (Gr-MDSCs) and dysfunctional T cells, while CIN low tumors were enriched in pro-inflammatory macrophages, IFN-responsive B cells, activated dendritic cells and CD4 + T helper cells [20]. In GIMGs-high samples, the majority of HLA genes were lower than those in GIMGs-low samples (Fig. 2D). In the GIMGs-high group, the expression of several immune checkpoint genes—including LAG3, TIGIT, PDCD1LG2, and CTLA4—were significantly elevated compared to the GIMGs-low group (Fig. 2E). These results indicated GIMGs were associated with an altered immune microenvironment and high expression of immune checkpoints in breast cancer.

### Identification of key GIMGs in breast cancer from TCGA and GEO database

**Fig. 3:**
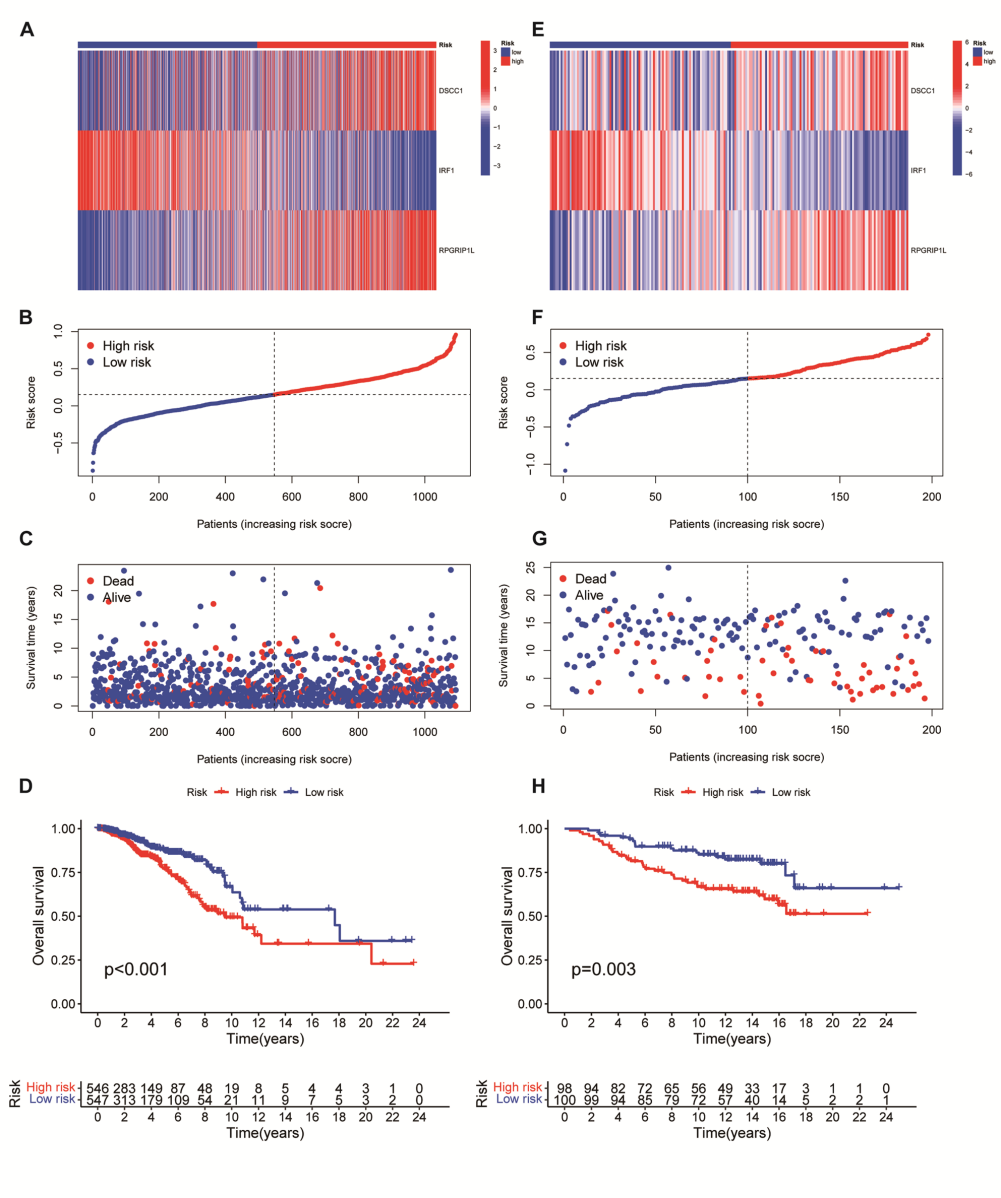
Identification of the prognostic value of the three GIMGs in the TCGA cohort and GEO cohort of breast cancer patients. (**A) **. The correlation of the expression of the three GIMGs (DSCC1, RPGRIP1L, and IRF1) and distribution of risk samples in TCGA cohort. (**B) **, The correlation of risk score and patient distribution in TCGA cohort. (**C) ** The correlation of risk score and survival status in TCGA cohort (**D**) Kaplan-Meier curve of OS in TCGA cohort. (**E) **, The correlation of the expression of the three GIMGs and distribution of risk samples in GEO cohort. (**F) **, The correlation of risk score and patient distribution in GEO cohort. (**G) **, the correlation of risk score and survival status in TCGA cohort. (**H) ** Kaplan-Meier curve of OS in the GEO cohort. ****p < 0.0001; ***p < 0.001; **p < 0.01; *p < 0.05

To find the mechanism how GIMGs affected prognosis in breast cancer, we further conducted a univariate COX regression analysis on the TCGA dataset to identify a minimal set of prognostic GIMGs and build a robust risk model, This analysis revealed that three genes, namely DSCC1, RPGRIP1L, and IRF1, were significantly correlated with patient prognosis (Fig. S1 A). Subsequently, LASSO Cox regression analysis was performed to obtain a GIMGs signature with an optimal λ value (Fig. S1B-C). The results demonstrated the effectiveness of the prognostic model built upon these three genes (Fig.S1D). Specific risk scores were computed for each sample utilizing a predefined formula. Graph showed increased expression of DSCC1, RPGRIP1L, and IRF1 in BRCA tumors compared with normal tissues form TCGA dataset (Fig. S1E). Survival analysis indicated that higher DSCC1 and RPGRIP1L expression was significantly related with shorter survival time in BRCA patients (Fig. S1F). Although IRF1 showed a protective effect in univariate analysis, it contributed to the model’s prognostic power in multivariate contexts. Furthermore, the results showed similar relationship between the three genes and the risk score (Fig. 3A), the risk score and patient distribution (Fig. 3B), and the risk score and survival status (Fig. 3C) in TCGA BRCA patients. As the Kaplan-Meier curves suggested, low-risk patients shared significantly superior OS to high-risk patients (Fig. 3D). The risk model’s feasibility was validated using the GEO dataset for external validation and accuracy assessment. Consistently, similar results were observed in GEO dataset (Fig. 3E-H).

**Fig. 4:**
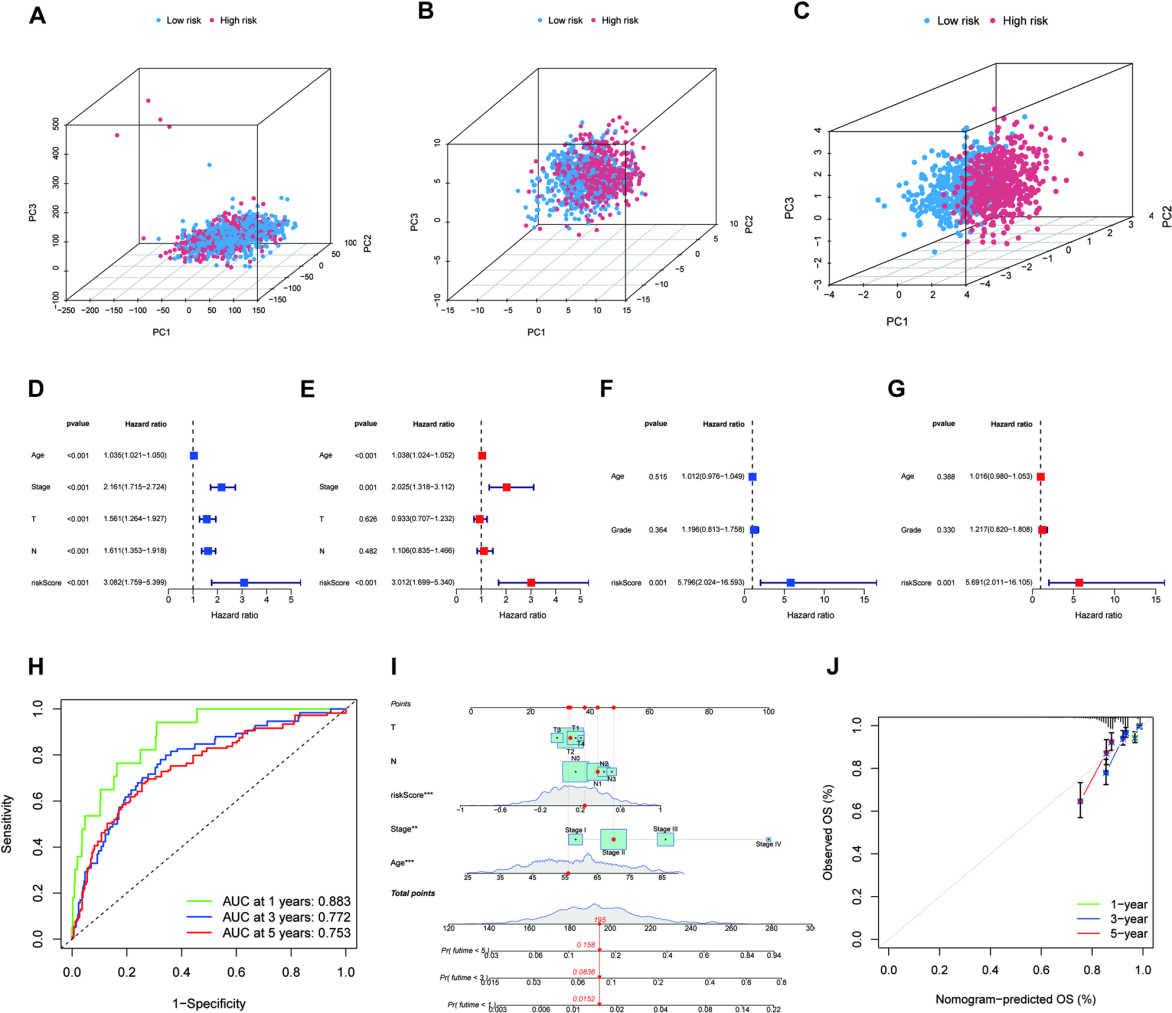
A nomogram for predicting the overall survival of breast cancer patients in TCGA and GEO dataset. (**A**) Principal component analysis of all genes according to risk score in TCGA cohort. (**B)** Principal component analysis of GIMGs according to risk score in TCGA cohort. (**C) ** Principal component analysis of the three genes signature according to risk score in TCGA cohort. *p < 0.05, **p < 0.01; ***p < 0.001; ns, not significant. Univariate Cox regression analysis (**D**) and multivariate Cox regression analysis (**E**) of clinical characteristics and risk score in breast cancer overall survival in the TCGA cohort. Univariate Cox regression analysis (**F**) and multivariate Cox regression analysis (**G**) of clinical characteristics and risk score in breast cancer overall survival in the GEO cohort. ROC curve of the risk score (**H**), a nomogram of the risk score (**I**), and calibration curves of this nomogram (**J**) for the prediction of 1,3,5-year OS of breast cancer patients in the TCGA cohort. ****p< 0.0001; ***p< 0.001; **p < 0.01; *p< 0.05

We then build a nomogram with these three genes to predict prognosis in BRCA patients. Initially, principal component analysis (PCA) was utilized to evaluate the grouping ability of the risk signature. Through the integration of whole-genome expression profiles with 74 GMIGs and a risk model, the analysis effectively segregated samples into two distinct risk groups, showcasing the risk model’s proficient stratification of samples (Fig. 4A-C). The results showed that risk model based on the three genes can better distinguish patients with different level of risk. Univariate and multivariate Cox regression analyses in TCGA and GEO datasets suggested that risk score was an independent prognostic factor for OS in breast cancer (Fig. 4D-G). The AUC values of risk score in TCGA cohort were 0.883 in 1-year, 0.772 in 3-year and 0.753 in 5-year OS. The creation of a nomogram provided a practical and quantitative method for predicting outcomes in individuals with breast cancer (Fig. 4I). Calibration curves were utilized to evaluate the reliability and consistency of the nomogram, highlighting a robust correlation between the predicted and actual survival rates (Fig. 4J). Consistently, similar results were observed in GEO dataset (Fig.S2A-C). These results indicated the three genes were the representative GIMGs in breast cancer. Moreover, the risk model based on the three genes demonstrates stable and reliable prognostic prediction capabilities for breast cancer patients.

### RPGRIP1L expression was increased in breast cancer and was associated with worse outcome in patients

**Fig. 5:**
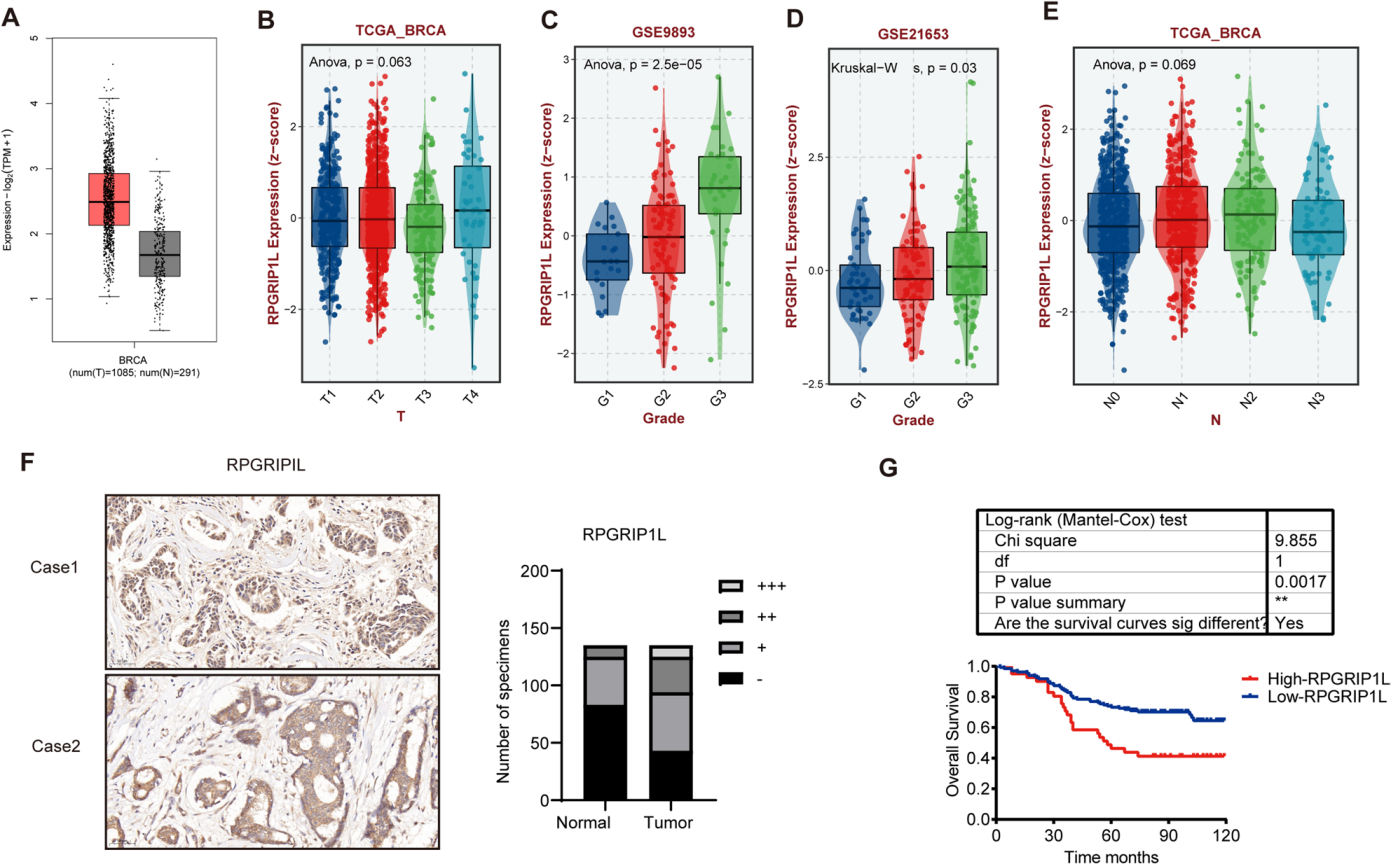
RPGRIP1L expression and functional analysis. (**A)**. Expression of RPGRIP1L in tumor and normal tissue from breast cancers. (**B**-**E)**. Correlation between RPGRIP1L expression and T, N stages and grades in TCGA and GEO datasets. (**F)**. Representative images of immunohistochemical (IHC) and quantitative analysis of RPGRIP1L in a human breast cancer tissue from 135 cases of breast cancer patients. (**G)**. Kaplan-Meier curve of OS of patients based on RPGRIP1L expression in tumor cells

Next, we focused on RPGRIP1L, which is less studied, to explore its biological function and mechanism in breast cancer. We then analyze the expression of RPGRIP1L in breast cancer from TCGA datasets. The result showed that RPGRIP1L mRNA expression was increased in breast cancer (Fig. 5A). We next explored the prognostic value of RPGRIP1L protein in GEO datasets. RPGRIP1L expression was positively correlated with grade (Fig. 5B-E). To further determine the protein expression of RPGRIP1L in breast cancer, we performed immunohistochemical (IHC) analysis in human breast cancer tissues from 135 cases of breast cancer patients. As a result, RPGRIP1L expression was highly expressed in tumor cells (Fig. 5F). And high expression of RPGRIP1L predicted poor prognosis in breast cancer patients (Fig. 5G).

### RPGRIP1L promoted tumor development and increased programmed death ligand 1(PD-L1) expression in breast cancer

**Fig. 6:**
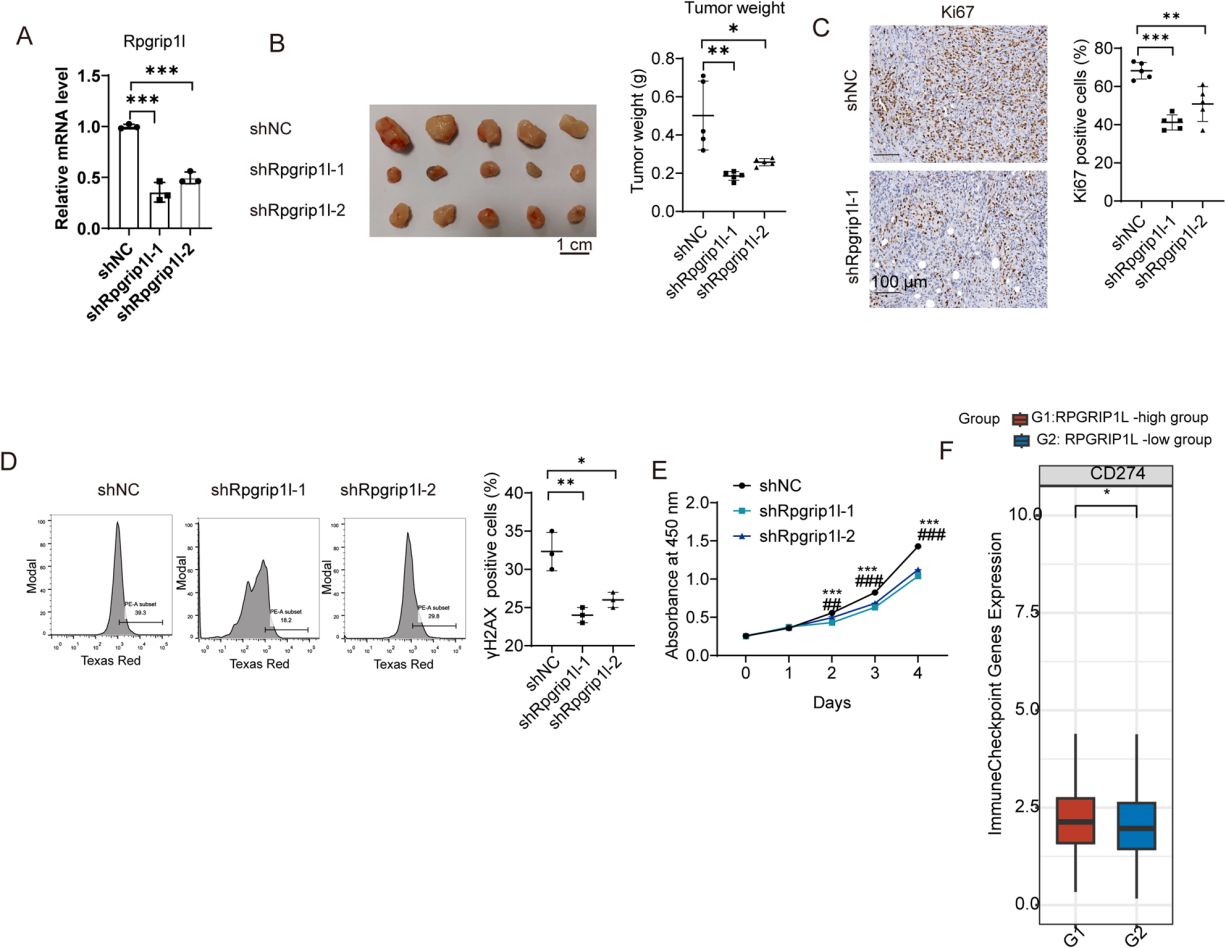
Effect of RPGRIP1L on tumor growth and immune microenvironment in breast cancer. (**A)** qPCR showing the knockdown efficiency of RPGRIP1L by shRNA in EO771 cells. (**B)** Representative tumor images and graph of tumor weights formed by shNC and shRPGRIP1L EO771 cells transplanted into the mammary fat pad of C57/B6L mice. (**C)** Ki-67 IHC staining in the tumor sections of mice. (**D)** Representative flow cytometry analysis of γH2AX expression and graph showing the percentage of γH2AX positive tumor cells in shNC and shRPGRIP1L EO771 cells. (**E)** CCK8 showing the cell proliferation. (**F)** The expression of PD-L1 in RPGRIP1L-high and RPGRIP1L-low groups of TCGA datasets. ****p < 0.0001; ***p < 0.001; **p < 0.01; *p < 0.05

To investigate the in vivo consequence of RPGRIP1L knockdown for cancer formation and metastasis, EO771 cells with RPGRIP1L knockdown by shRNA or shNC (Fig. 6A), and then directly transplanted into the mammary fat pad of C57/B6L mice. RPGRIP1L knockdown significantly decreased the tumor burden of breast cancer (Fig. 6B). Ki-67 IHC staining showed decreased tumor proliferation after RPGRIP1L knockdown (Fig. 6C). In vitro, Flow cytometry analysis of γH2AX expression, an indicator of the presence of DNA damage, showed increased genomic stability after RPGRIP1L knockdown (Fig. 6D). CCK8 results showed tumor cells proliferation was decreased after RPGRIP1L knockdown in tumor cells (Fig. 6E). We then investigated the role of RPGRIP1L on tumor immune checkpoints. Previous studies have reported that PD-L1 (CD274) was a very important immune checkpoint in breast cancer. And the result showed that PD-L1 was higher in the RPGRIP1L-high group (Fig. 6F). These results indicated a cancer-promoting role of RPGRIP1L in breast cancer via both direct effect on tumor cells and modulating immune checkpoint.

### RPGRIP1L increased PD-L1 expression via upregulated Hedgehog signaling pathway

**Fig. 7:**
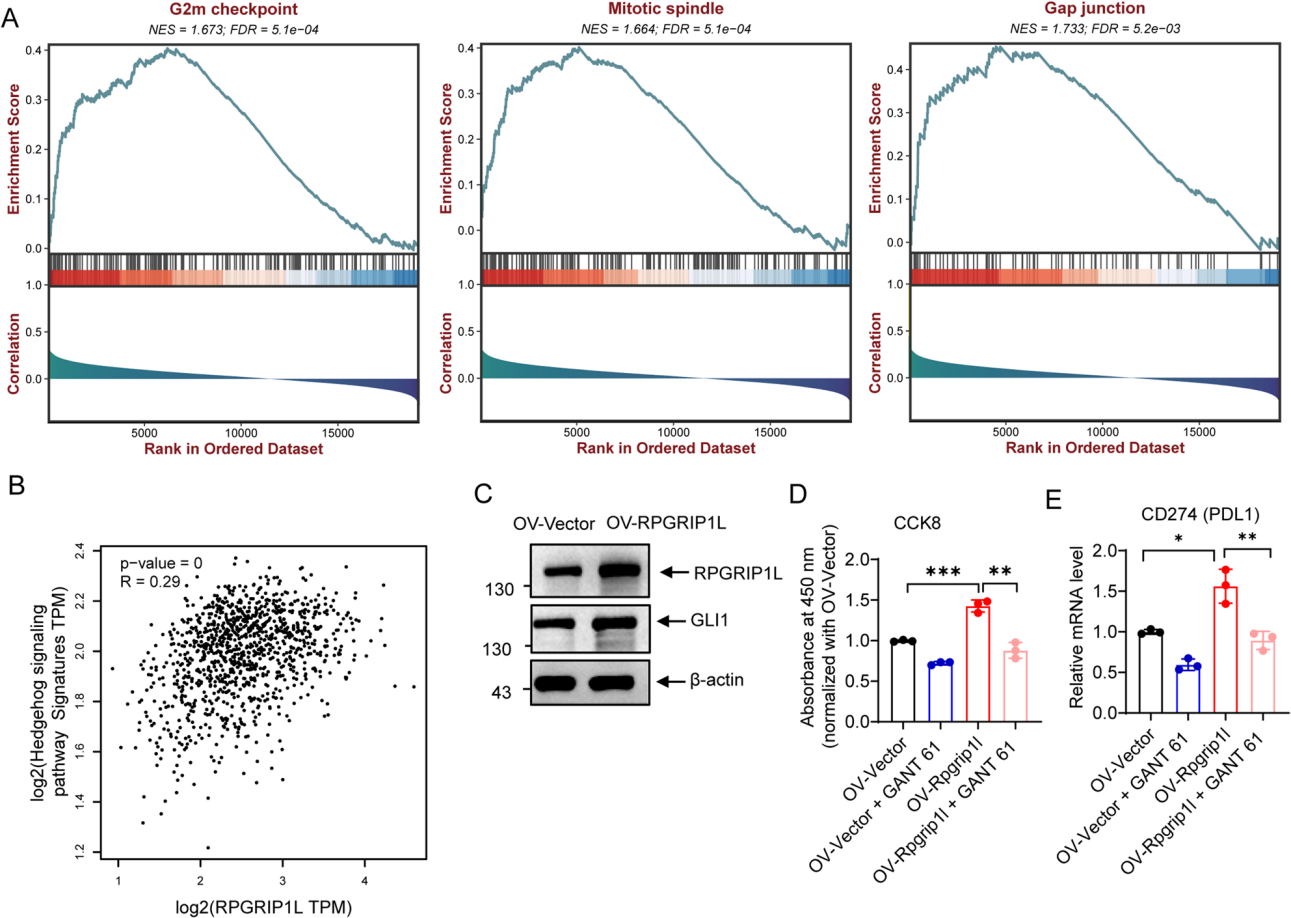
Activation and effect of Hedgehog signaling pathway in RPGRIP1L high expressing breast cancer. **(A)** GSEA of RPGRIP1L in TCGA-BRCA datasets. (**B)** Correlation analysis of RPGRIP1L with Hedgehog signaling pathway analyzed on GEPIA2 (http://gepia2.cancer-pku.cn/#index). (**C)** Western blot showing the protein expression of RPGRIP1L and GLI1 in tumor cells with RPGRIP1L overexpression. (**D)** CCK8 of the cells treated with Hedgehog signaling pathway inhibitor GANT61. (**E)** qPCR showing the mRNA expression of CD247 (PD-L1). ****p < 0.0001; ***p < 0.001; **p < 0.01; *p < 0.05

To explore the molecular basis by which RPGRIP1L promotes BRCA development, Gene set enrichment analysis (GSEA) showed G2M checkpoint, mitotic spindle and gap junction were enriched in RPGRIP1L high expression samples in BRCA (Fig. 7A). As we know that, RPGRIP1L promotes Hedgehog signaling pathway activation [21]. The correlation between RPGRIP1L and Hedgehog signaling activity was analyzed using RNA-seq data from the TCGA-BRCA cohort. GSEA was performed using the Hedgehog signaling gene set from the Molecular Signatures Database (MSigDB). Correlation analysis showed RPGRIP1L high expression was positively associated with Hedgehog signaling pathway activation (Fig. 7B). In tumor cells with RPGRIP1L overexpression, we found GLI1 protein expression was increased (Fig. 7C). Activated GLI1 has been reported to trigger the transcription of PD-L1 and promotes immunoscape in hepatocellular carcinoma [22]. We found inhibitors of Hedgehog signaling pathway GANT61 suppressed tumor proliferation and PD-L1 expression in RPGRIP1L overexpressing breast cancer cells (Fig. 7D-E). These data suggested that RPGRIP1L upregulated Hedgehog signaling pathway, then increased cell proliferation and PD-L1 expression in BRCA.

### RPGRIP1L expression and tumor mutation burden in TCGA cohort

**Fig. 8:**
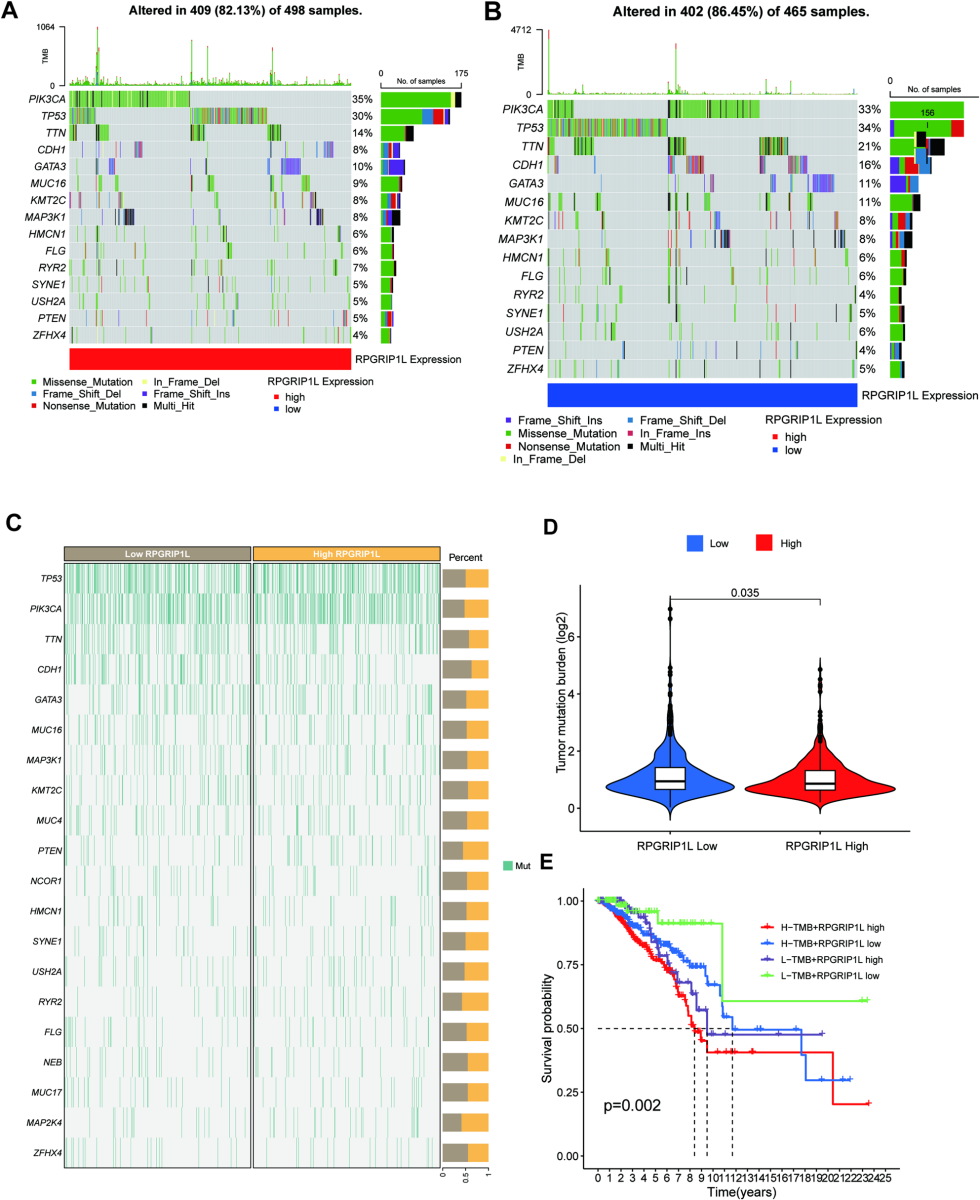
Tumor mutation analysis of RPGRIP1L. (**A** and **B)**. Waterfall plots presented depiction of the mutation data in RPGRIP1L-high and low group. (**C)**. Mutation frequencies in RPGRIP1L-high and low group. (**D)**. Correlation between RPGRIP1L expression and tumor mutation burden (TMB). (**E)**. Patient survival with high and low TMB combined with RPGRIP1L expression

It has been reported that tumor mutation burden (TMB) was related to the sensitivity of immunotherapy. We further did the analysis the relationship between RPGRIP1L and TMB. Waterfall plots presented a visual depiction of the mutation data (Fig. 8A-B), and we found similar mutant genes and significant difference of tumor mutation burden between RPGRIP1L low and high samples (Fig. 8C-D). Then, combining RPGRIP1L and TMB, we found that patients with high TMB from the RPGRIP1L high group suffered the worst prognostic outcome (Fig. 8E).

## Discussion

Chromosomal instability is a hallmark of human cancer. It is associated with occurring, development, metastasis, and therapeutic resistance in breast cancer. How cancer cells maintain genomic instability is an important question to be answered. Clarifying the mechanisms controlling the stability of genomes can reveal central insights into the mechanism for tumor development. In this study, we explored the genetic determinants of genomic instability in breast cancer. We also established a set of genomic instability controlling genes (DSCC1, RPGRIP1L, and IRF1) and a new risk model in breast cancer. This might provide a conceptual platform for the identification of genomic instability related targets in breast cancer treatment. Specifically, we identified RPGRIP1L, the main genomic instability maintaining gene promotes tumor cell growth and immune escape by activating Hedgehog signaling pathway.

In addition to generating genomic heterogeneity that acts as a substrate for natural selection, chromosomal instability also promotes inflammatory signaling. Therefore, these multipronged effects distinguish chromosomal instability as a central driver of tumor evolution and as a genomic source for the crosstalk between the tumor and its microenvironment [14]. The interaction between genome instability and immune is complex. This tumor cell-intrinsic effect potentiates activation or inhibition of infiltrating immune cells. It has been reported that Poly (ADP-ribose) polymerase 1, the key nuclear sensor of DNA damage, links the genome instability to the inhibition of antiviral immunity [19]. CDK7 inhibition impairs cell cycle and DNA replication and induces genome instability, while its inhibition by YKL-5-124 provokes a robust immune program in small cell lung cancers [23]. Nonetheless, genome instability in tumor cells can affect immune responses, although the mechanism responsible for different immune changes might depend on specific genome controlling genes.

Samples with upregulated RPGRIP1L signaling showed an increase in immune checkpoint molecules like PD-L1, which is associated with better responses to anti-PD-1 therapy. Consistent with previous reports on the role of RPGRIP1L in forebrain midline structures and facial development [24], RPGRIP1L was found to overactivate the Hedgehog signaling pathway in breast cancer, as evidenced by increased GLI1 expression in this study. Many studies have highlighted the Hedgehog pathway and signaling other pathways as critical for breast cancer cell proliferation, survival, differentiation, cancer stem cell maintenance, and therapy resistance [25]. Notably, GLI1 has been shown to promote PD-L1 transcription by directly binding to its promoter [22]. Using a GLI1 inhibitor, we demonstrated that RPGRIP1L enhanced cell proliferation and PD-L1 expression in BRCA. However, whether GLI1 plays the dominant role remains a question for future research. Moreover, TMB correlates with response to immune checkpoint inhibitors in many solid tumor types. Phase II KEYNOTE-158 study shows that the anti-PD-1 antibody pembrolizumab was granted approval for treating patients with advanced solid tumors and TMB ≥ 10 mutations per megabase [26]. In our study, patients with high TMB from the RPGRIP1L high group suffered the worst prognosis. Whether RPGRIP1L upregulates TMB or RPGRIP1L correlates with good response to anti-PD-1 therapy need further study.

Although our study is the systemic analysis of genomic instability maintaining genes in breast cancer. There are some limitations need to be acknowledged. Firstly, the gene sets are chosen based on the reported genes associated with genomic instability identified in mice cells, therefore, genes only expressed in human might be missed. Secondly, the specific molecular mechanism of RPGRIP1L controlling genome instability was undetermined in breast cancers. Thirdly, the mechanism between genomic instability and tumor immunotherapy was elusive. Therefore, more basic and translational researches are needed to refine the discoveries in our study.

## Conclusion

In this study, we found the GIMGs in breast cancer and set a three-gene risk score to predict prognosis in breast cancer patients. Moreover, we found RPGRIP1L, one of the three gene, promoted breast cancer progression via Hedgehog-PD-L1 axis, which indicated that RPGRIP1L may be a new target for breast cancer treatment.

## Supplementary Information

Supplementary Material 1.

Supplementary Material 2.

Supplementary Material 3.

Supplementary Material 4.

## Data Availability

The data that support the findings of this study are available from the corresponding author upon reasonable request.
