# Supplementary figures and images for: Identification of RPGRIP1L as an instability-maintaining gene to drive tumor growth and PD-L1 expression via Hedgehog signaling in breast cancer
Source: BMC Cancer. 2025 Dec 30;26:165. doi: 10.1186/s12885-025-15500-2 (PMC12866045; doi:10.1186/s12885-025-15500-2)

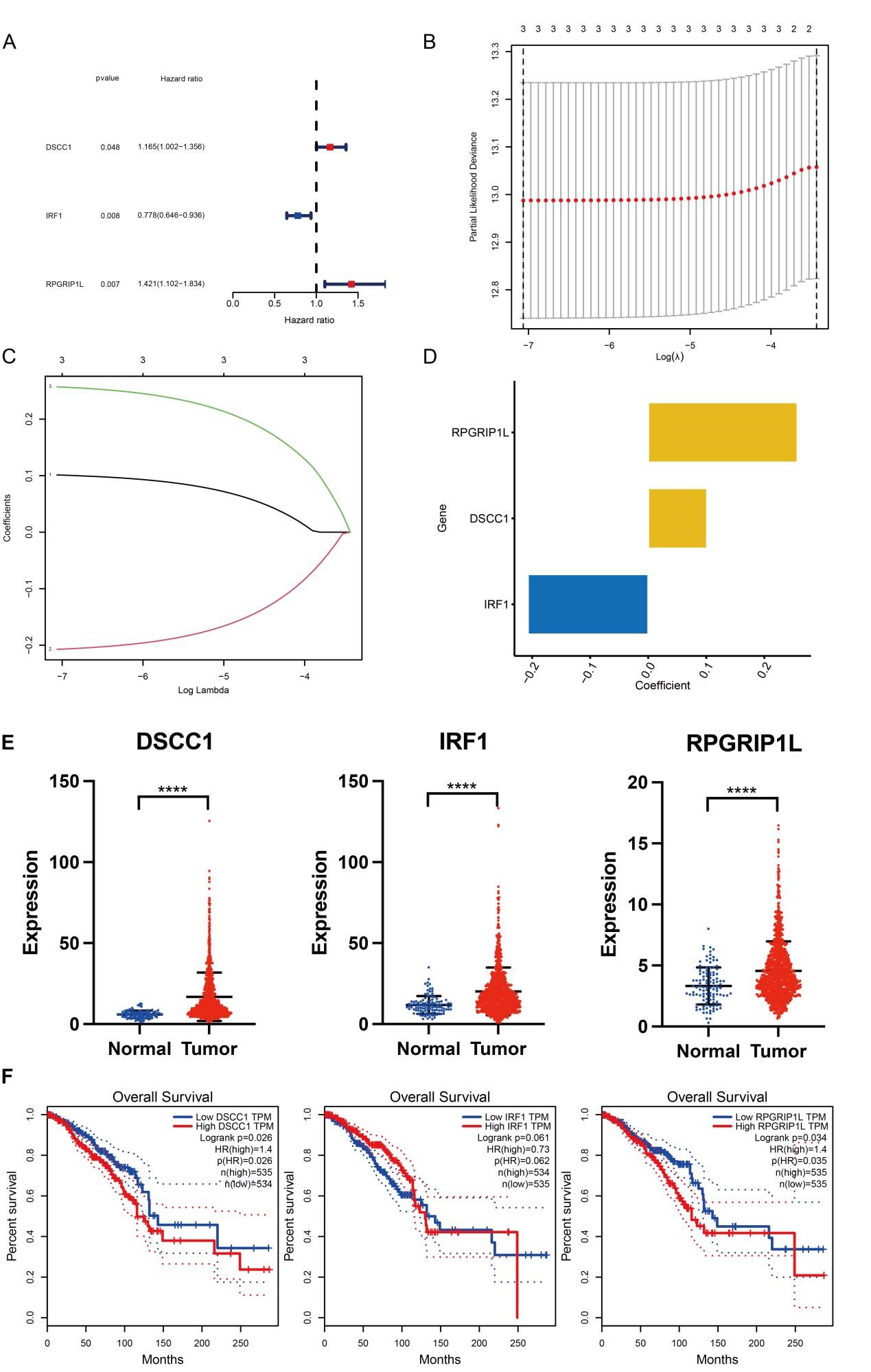

Supplement: Supplementary file 1 — Supplementary Material 1. [file 12885_2025_15500_MOESM1_ESM.jpg]

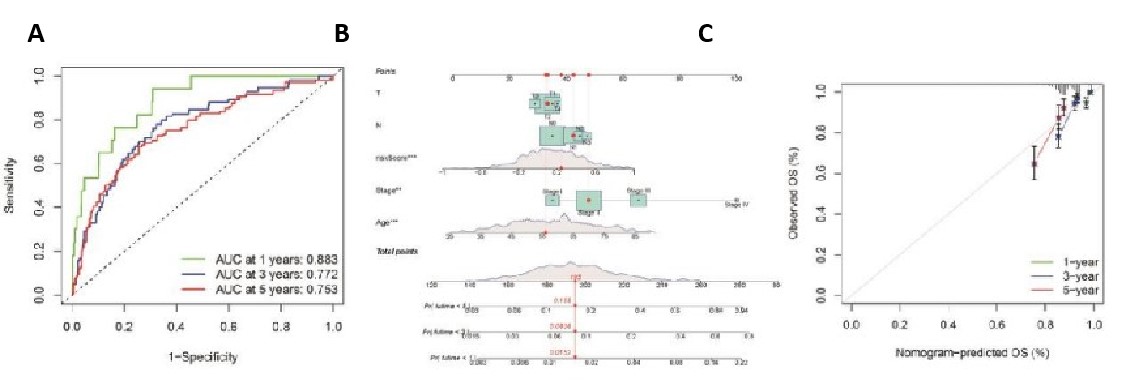

Supplement: Supplementary file 2 — Supplementary Material 2. [file 12885_2025_15500_MOESM2_ESM.jpg]
