# Supplementary material for: Identification of RPGRIP1L as an instability-maintaining gene to drive tumor growth and PD-L1 expression via Hedgehog signaling in breast cancer
Source: BMC Cancer. 2025 Dec 30;26:165. doi: 10.1186/s12885-025-15500-2 (PMC12866045; doi:10.1186/s12885-025-15500-2)
Supplement: Supplementary file 3 — Supplementary Material 3. [file 12885_2025_15500_MOESM3_ESM.docx]

Supplementary Figure Legends

**Figure S1. Recognition of three GIMGs with prognostic value in breast cancer**. (A). Forest plot of GIMGs obtained by a univariate Cox regression analysis. (B and C). LASSO expression was performed. (B) showing the cross-validation plot for the penalty term, and (C) showing plots for LASSO expression coefficients of the GIMG. (D). The coefficient of three GIMGs. (E). Relative mRNA expression of the three GIMGs in normal breast and tumors. (F). Overall Survival of the three GIMGs from breast cancer patients. Samples of breast cancers were from TCGA. ****p< 0.0001; ***p< 0.001; **p < 0.01; *p< 0.05.

**Figure S2. A nomogram for predicting the overall survival of breast cancer patients in GEO dataset**

1. .ROC curve of the risk score ,(B). a nomogram of the risk score, and(C).

calibration curves of this nomogram for the prediction of 1,3,5-year OS of breast cancer patients in the GEO cohort.****p< 0.0001; ***p< 0.001; **p < 0.01; *p< 0.05
