# Supplementary material for: Identification of RPGRIP1L as an instability-maintaining gene to drive tumor growth and PD-L1 expression via Hedgehog signaling in breast cancer
Source: BMC Cancer. 2025 Dec 30;26:165. doi: 10.1186/s12885-025-15500-2 (PMC12866045; doi:10.1186/s12885-025-15500-2)

Figure 1. Western blot showing the protein expression of RPGRIP1L in tumor cells with RPGRIP1L overexpression.

Marker OV-Vector OV- RPGRIP1L

130KD

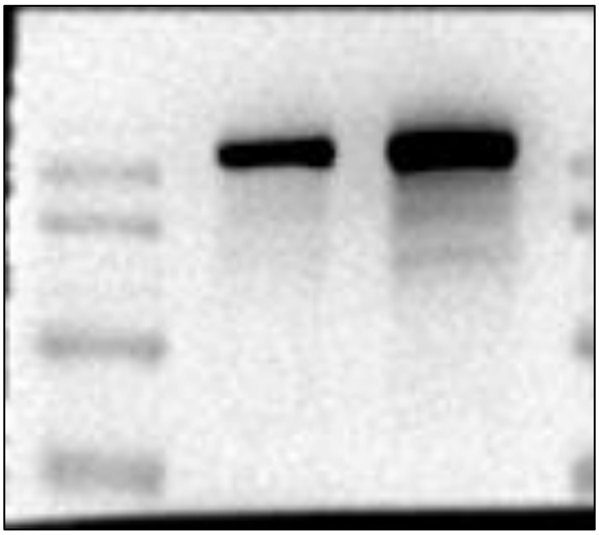

Figure 2. Western blot showing the protein expression of GIL1 in tumor cells with RPGRIP1L overexpression.

Marker OV-Vector OV- RPGRIP1L

130KD

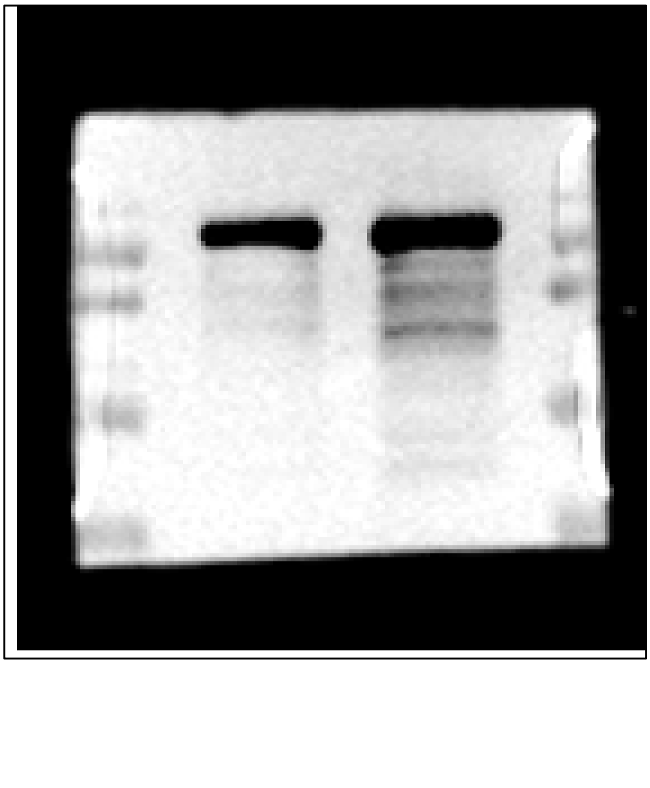

Figure 3. Western blot showing the protein expression of β-actin in tumor cells with RPGRIP1L overexpression.

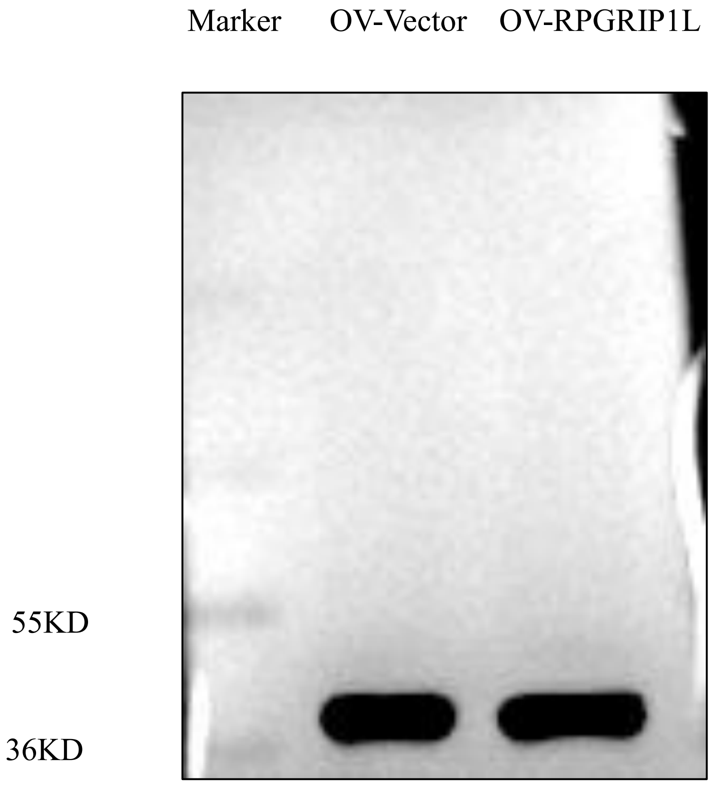

Supplement: Supplementary file 4 — Supplementary Material 4. [file 12885_2025_15500_MOESM4_ESM.pdf]
